# Bridging community and clinic through digital health: Community-based adaptation of a mobile phone-based heart failure program for remote communities in Uganda

**DOI:** 10.1186/s44247-023-00020-5

**Published:** 2023-06-16

**Authors:** Sahr Wali, Isaac Ssinabulya, Cinderella Ngonzi Muhangi, Jenipher Kamarembo, Jenifer Atala, Martha Nabadda, Franklin Odong, Ann R. Akiteng, Heather Ross, Angela Mashford-Pringle, Joseph A. Cafazzo, Jeremy I. Schwartz

**Affiliations:** 1 Institute of Health Policy, Management and Evaluation, Dalla Lana School of Public Health, University of Toronto, Toronto, ON Canada; 2 Centre for Digital Therapeutics, Toronto General Hospital, University Health Network, R. Fraser Elliott Building, 4th floor, 190 Elizabeth St, Toronto, ON M5G 2C4 Canada; 3 Initiative for Integrated Management of Non-Communicable Diseases, Kampala, Uganda; 4 Uganda Heart Institute, MulagoNational Referral Hospital, Kampala, Uganda; 5 Gulu Regional Referral Hospital, Gulu, Uganda; 6 Lira Regional Referral Hospital, Lira, Uganda; 7 Ted Rogers Centre for Heart Research, University Health Network, Toronto, ON Canada; 8 Institute of Medical Sciences, Faculty of Medicine, University of Toronto, Toronto, ON Canada; 9 Peter Munk Cardiac Centre, University Health Network, Toronto, ON Canada; 10 Dalla Lana School of Public Health, Waakebiness-Bryce Institute for Indigenous Health, University of Toronto, Toronto, ON Canada; 11 Institute of Biomedical Engineering, University of Toronto, Toronto, ON Canada; 12 Department of Computer Science, University of Toronto, Toronto, ON Canada; 13 Section of General Internal Medicine, Yale University School of Medicine, New Haven, USA

**Keywords:** digital health, heart failure, community-based research, two-eyed seeing, user-centered design

## Abstract

**Background:**

In Uganda, limited healthcare access has created a significant burden for patients living with heart failure. With the increasing use of mobile phones, digital health tools could offer an accessible platform for individualized care support. In 2016, our multi-national team adapted a mobile phone-based program for heart failure self-care to the Ugandan context and found that patients using the system showed improvements in their symptoms and quality of life. With approximately 84% of Ugandans residing in rural communities, the Medly Uganda program can provide greater benefit for communities in rural areas with limited access to care. To support the implementation of this program within rural communities, this study worked in partnership with two remote clinics in Northern Uganda to identify the cultural and service level requirements for the program.

**Methods:**

Using the principles from community-based research and user-centered design, we conducted a mixed-methods study composed of 4 participatory consensus cycles, 60 semi-structured interviews (SSI) and 8 iterative co-design meetings at two remote cardiac clinics. Patient surveys were also completed during each SSI to collect data related to cell phone access, community support, and geographic barriers. Qualitative data was analyzed using inductive thematic analysis. The Indigenous method of *two-eyed seeing* was also embedded within the analysis to help promote local perspectives regarding community care.

**Results:**

Five themes were identified. The burden of travel was recognized as the largest barrier for care, as patients were travelling up to 19 km by motorbike for clinic visits. Despite mixed views on traditional medicine, patients often turned to healers due to the cost of medication and transport. With most patients owning a non-smartphone (*n* = 29), all participants valued the use of a digital tool to improve equitable access to care. However, to sustain program usage, integrating the role of village health teams (VHTs) to support in-community follow-ups and medication delivery was recognized as pivotal.

**Conclusion:**

The use of a mobile phone-based digital health program can help to reduce the barrier of geography, while empowering remote HF self-care. By leveraging the trusted role of VHTs within the delivery of the program, this will help enable more culturally informed care closer to home.

**Supplementary Information:**

The online version contains supplementary material available at 10.1186/s44247-023-00020-5.

## Introduction

Across sub-Saharan Africa (SSA), the burden of non-communicable diseases (NCDs) has grown significantly, whereby it is estimated that NCDs will be the leading cause of death by 2030 [1, 2]. Among chronic diseases, heart failure (HF) has been found to be responsible for up to 30% of all NCD-related deaths and 7% of all hospital admissions in SSA [1, 3–7]. Given the progressive nature of the condition, HF is often characterized by its recurrent exacerbations, leading patients to experience chronic symptoms of fatigue and shortness of breath, punctuated by sporadic clinical decompensations [6, 7]. Despite the severity of the disease, these symptoms can often be addressed, if patients are given the tools to support their ability to engage in self-care (e.g., managing diet, diuretics, other medications) [8, 9]. However, with many communities in SSA currently living in poverty, complex challenges related to the disparities in their social determinants of health (SDH) (i.e., housing, income, food security, access to quality care), have limited the benefits of self-care to be realized [2, 10]. With this, to better support the unique needs of patients living in SSA, self-care strategies need to accommodate both the clinical and social factors impacting their well-being.

Recognizing the interplay between poverty and health care access, digital health has emerged as an affordable avenue to improve the equitable distribution of health services [11–13]. On a global scale, mobile phone use has become nearly universal, whereby many individuals who have never used traditional landlines are now using mobile phones on a daily basis [14]. For example, in Uganda, a recent report found that approximately 74% of all households own a mobile phone [15]. With many Ugandan communities facing geographic barriers to care, digital health can serve as a low-cost approach to provide clinical expertise and remote support [13–15]. However, despite the benefits of these tools, it is important to recognize that many digital health studies have found mixed success in improving population health outcomes within low-resource settings [13, 14]. Given that the majority of these tools have been designed for use in high resource settings, this discrepancy in health improvement can be largely linked to the lack of contextual and cultural considerations incorporated within digital tool design [16]. Interventions that fail to integrate or understand the unique challenges faced by the community continue to be underused and/or abandoned by communities during the early stages of adoption [14–16]. To improve the uptake and impact of a digital health program, the local needs and capabilities of the community would need to be integrated within the design of an intervention.

### Medly Uganda: Digital health program for remote heart failure care

With a growing evidence base supporting the beneficial use of digital health, in 2016, the Uganda Heart Institute (UHI), University Health Network (UHN), and Yale University established a collaboration to adapt a mobile phone-based HF program for the Ugandan setting. The original program, known as Medly, was first developed, and tested in Toronto, Canada, with an interdisciplinary team of HF specialists, engineers, designers, and patients, to transform the way HF patients manage their condition [17–19]. With Medly, a class II Health Canada cleared software-as-a-medical-device, patients use a smartphone, weight scale, and blood pressure cuff to record daily physiologic readings and report and respond to symptom queries [19, 20]. This data runs through a rules-based algorithm, and provides patients with individualized self-care instructions, while simultaneously alerting clinicians when there are changes in the patient’s health status via the clinician dashbard [18, 19]. Recognizing the contextual differences between the Canadian and Ugandan setting (i.e., resource availability, cultural context, language), the Medly Uganda collaborative team conducted a mixed methods study, composed of in-depth stakeholder interviews (i.e., clinicians, nurses, patients), patient surveys, and in-person usability testing, to adapt the system to the local environment [21]. System adaptations included: 1)used existing open-source mHealth system, managed by Uganda Ministry of Health (i.e., FamilyConnect), to develop patient-facing app; 2) deployed service of Medly Uganda system using Unstructured Supplementary Service Data (USSD) technology commonly used across Uganda; 3) removed home-based vital signs from rules-based algorithm due to lack of weight scales and blood pressure cuffs in the home setting; 4) removed diuretic dose adjustment from self-care advice due to potential patient safety concerns (e.g., lack of home-based vital signs or standardized laboratory testing); and 5) translated system to Runyankole and Luganda. Further details regarding the system adaptations and methods of evaluation for the Medly Uganda platform have been previously described [21, 22]. To evaluate the impact of the Medly Uganda program on patient outcomes, a single-arm, pilot clinical trial (ClinicalTrials.gov Identifier: NCT04426630), based at UHI in Kampala, Uganda, was recently completed [23]. With the use of tailored self-care advice and local nurse support, patients were able to use simple feature phones to show improvements in both their HF self-care and health-related quality of life [23].

### Focus on geographic context: Rural vs urban poverty

In Uganda, over 84% of the population currently resides in rural communities, yet the majority of health care resources are located within urban centers [24, 25]. With these conditions, our team recognizes that patients in the initial Medly Uganda pilot trial had the geographic benefit of residing near the capital city or the financial means to travel to UHI [15, 24]. Given the challenge of rural care access, the Medly Uganda program can be used as a supportive tool to empower patient self-care, while reducing the burden of travel, for communities that are often overlooked. However, given the complexity of the rural environment, several considerations related to the community’s clinical sources, mobile network connection, cultural practices and traditional foods, would need to be integrated within the program specifications [24, 25]. With a combination of various context-specific methodologies, the objective of this mixed methods study was to work in partnership with two rural communities to adapt the Medly Uganda program to their local context. This paper will outline 1) the partnership building process used to co-develop the project priorities and study design, 2) the community-based needs assessment to better understand the contextual challenges and unique barriers affecting HF care and 3) the iterative process of identifying and integrating the program adaptations to improve the effectiveness and relevance of the program to the local communities.

## Methods

### Setting

UHI, based in Kampala, currently serves as the sole provider of advanced cardiovascular diagnosis and treatment in Uganda’s public health sector [26]. Given the level of expertise, UHI also oversees cardiac care at several regional hospitals throughout the country [26]. Specifically, as part of the national rheumatic heart disease (RHD) program, UHI works with four clinics in the Central, Western, and Northern regions of Uganda (i.e., Lubowa, Mbarara, Gulu, and Lira) [26]. With the shortage in cardiologist availability, UHI been limited in its ability to provide local outreach visits in the more remote communities [26, 27]. To address this issue, this study initiated a partnership with the Gulu and Lira cardiac clinics to better understand the contextual challenges faced in the community, as well as the cultural and service level needs to adapt the Medly Uganda program.

This study was conducted between January 26, 2021, and June 30, 2022, at the Gulu Regional Referral Hospital, and Lira Regional Referral Hospital, located 332 km and 338 km from the capital city of Kampala respectively. Study approval was obtained by the Makerere University School of Medicine Research Ethics Committee (Mak-SOMREC-2021–63) and the UHN Research Ethics Board (ID #: 20–6022).

### Study design

This study used a mixed methods participatory social justice design for the remote clinic adaptation of the Medly Uganda program [12, 28, 29]. The principles of user-centered design (UCD) and community-based participatory research (CBPR) were leveraged to ensure the systematic consideration of community needs throughout all stages of the design life cycle [21, 29, 30]. Both the UCD and CBPR frameworks use a relational approach to research to empower community involvement and facilitate community-oriented change. With Western research often solely fixating on the physical state of individual well-being, a series of Indigenous-based methodologies were used to better integrate the values of CBPR within the study procedures [29, 31]. Many Indigenous methods are centered on the principles of community and interconnectedness, whereby an individual’s wellness is influenced by the balance between their mental, physical, spiritual and emotional well-being [31, 32]. In this study, Indigenous methods developed in Canada and Australia were used to embrace the holistic process of self-reflection, and better understand the complexity of cultural context within multiple worldviews [12, 31]. The reflexive tenet of “Nothing About Us Without Us”, focused on partnership, mutual learning, and informed co-design, was also used as the theoretical paradigm for the study [32]. This manuscript provides an overview of the core methodology used within this study and a comprehensive description of the results from the community-based adaption of the Medly Uganda program. The full protocol describing the details of each study phase will be described elsewhere.

### Study procedures

In alignment with the principles from CBPR, Phase 1 of this study was centered on partnership building. To ensure respectful engagement strategies were used throughout all study phases, the Intervention and Research Readiness Engagement and Assessment of Community Health Care (I-RREACH) tool was utilized [12]. The I-RREACH tool is a community-based engagement and assessment resource, comprised of five core components to support the implementation of health interventions in low-resource environments 1) Indigenous participatory consensus cycles, 2) community profile tool, 3) key informant interview guide, 4) focus group guide and 5) participant evaluation survey.

With local guidance from the UHI team, the Northern clinics in Lira and Gulu were identified as the initial sites for community outreach. Both Northern clinics expressed a strong interest in the collaboration, as challenges related to resource limitations significantly impacted their care delivery. To co-develop the project priorities and study approach, a series of engagement sessions with key clinic and community stakeholders were conducted using the I-RREACH tool’s participatory consensus cycles. The first cycle involved an introductory discussion to explore the local perspective regarding the community’s current care priorities and the proposed research approach. At the end of this cycle, participants completed the adapted I-RREACH clinic profile and community demographic survey (Supplementary file 1) [12]. The second cycle served as the validation session to confirm each stakeholders’ views regarding the study objectives and design. This is known as *member checking.* This cycle was complemented by an in-person site visit by a member from the UHI team (MN), using the adapted I-RREACH tool’s community profile tool, for a deeper understanding of the local environment [12]. Finally, the third cycle was used as the final member checking to validate the overall research approach, as well as to gain stakeholder input on the practical aspects of the study regarding recruitment and community outreach [12].

Once the study approach was approved by all stakeholders, an iterative needs assessment with a convenience sample of HF patients, clinicians, and village health teams (VHTs) was conducted in Phase 2. Unique to the Ugandan context, VHTs were established by the Ministry of Health (MOH) as a collaborative initiative to help bridge the gap between communities and health facilities. Given their role in providing home visits, education, and clinic referrals, VHTs were included throughout all phases of this study [33]. In accordance with the guidance from the local stakeholders, participants were recruited using clinic referrals and VHT-led community outreach initiatives. Once written informed consent was obtained, all participants completed a yarning-style semi-structured interview and the adapted I-RREACH tool’s participant evaluation survey (Supplementary file 2) [12, 34]. Patients were also asked to complete a demographic survey (Supplementary file 3) to better understand the interdependent factors influencing their care management. This included elements related to their lifestyle, access to care, community support, medication availability and cell phone access. All participants were offered the option to conduct the interview in Acholi, Luo, Runyankole, Lango, or English. Interview transcripts were deidentified and transcribed verbatim using conventional content analysis and the Indigenous method of *two-eyed seeing* [35]. The process of *two-eyed seeing*, developed by Mi’kmaq Elder Albert Marshall, is defined as a metaphor for negotiating between two cultures [35]. By learning to see the strengths of Indigenous (in this case Ugandan) knowledge from one eye and the strengths of Western knowledge from the other, this reflexive approach to inquiry provides a conceptual framework to equitably embrace multiple perspectives within its own value. To support the application of this method, three authors were involved in the data analysis process at each site (Gulu: CN, JK, SW; Lira: CN, JA, SW). All transcripts were first analyzed independently by each team member, then five collaborative analysis sessions were held to develop themes reflective of both the Ugandan and Western worldview. To examine the thematic findings in combination with the survey data, Eakin and Gladstone’s “value-adding” approach was also utilized [36]. Two collaborative analysis sessions were held to discuss the contextual realties described within the survey data and its significance in relation to the themes generated. This process of value-adding provided an enriched interpretation of the analysis, as study data was not only evaluated in its ability to generate knowledge, but also its ability to reconceptualize the community’s evolving contextual narrative.

In Phase 3, the qualitative and quantitative data from the previous phase were used to adapt the Medly Uganda program for each site. Design requirements were identified in collaboration with the clinic and community stakeholders through a series of co-design meetings. The core study team (i.e., Lead Gulu nurse, Lead Lira nurse, UHI Leads, translator) was first consulted to discuss the study findings and identify the appropriate mode to engage with the community stakeholders (i.e., VHTs, parish coordinators). Following this, weekly meetings were held with the core study team to co-develop and refine the adapted program features. With the support from the Lead Nurse at each site, a series of co-design workshops with the VHTs were conducted. These sessions served as an opportunity to co-develop the VHT role within the refined Medly Uganda program workflow, as well as to obtain feedback on the adapted program features. Throughout this phase, the two-eyed seeing approach was used to ensure the local perspective was recognized for the co-development of the design features.

## Results

### Phase 1: Partnership building and learning

Four engagement sessions were conducted with each community site to co-develop the project priorities and overall study approach (Supplementary file 4: Appendix A). In Cycle 1, both clinic and community stakeholders (Gulu *n* = 7, Lira *n* = 6) participated in a virtual engagement session to introduce the project collaboration. Throughout the session, a focus on both HF and RHD was proposed to support a wider audience of cardiac patients. Clinic stakeholders emphasized the importance of using the Medly Uganda program to support a more seamless connection with the cardiac specialists in Kampala. Using the I-RREACH tool’s clinic profile, both sites recognized transport costs, limited drug supply, long wait times and lack of personnel or family support as the core barriers limiting patients from attending clinic visits. With respect to cardiac health support in the clinic, the Lira site indicated that the hospital tried to adjust its budget to cater for some cardiac medicines, whereas in Gulu, the clinic expected the government hospital to provide nursing support, but no concrete commitment has been made yet (Supplementary file 4: Appendix B). Within the community demographic survey, the use of radio talk shows was highlighted as the main avenue to promote health or wellness, and VHTs or community health workers were noted as the core individuals providing in-community support (Supplementary File 4: Appendix C).

In Cycle 2, the Lead Nurse at each clinic provided a thorough walk-through of the clinic workflows and the current challenges experienced by both patients and healthcare staff. In Gulu, patients were primarily seen in the cardiac clinic, whereas in Lira, the RHD clinic provided nurse-led cardiac care services from Monday to Friday, and the Out-patient department (OPD) medical clinic provided physician-led care for a range of medical conditions on Tuesday. However, despite the significant number of clinicians available (*n* = 8) at the OPD clinic, patients were often referred to the RHD clinic for clinical investigation. Across both sites, care continuity was recognized as a significant challenge, as many patients failed to return to clinic once their symptoms improved (Table 1). With a lack of community sensitization regarding heart health management and the limited services available at each clinic, identifying opportunities to incentivize self-care were pivotal to the adoption of the Medly Uganda program.

**Table 1:** Core findings from site visit’s guided discussion on heart health priorities

Characteristic	Gulu Clinic	Lira Clinic
How important is heart health to the patients seen in the clinic and community?	•Big gap in continuity of care •Majority of patients do not prioritize heart health •After improvement in symptoms, most patients do not return to clinic	•Awareness in the community is low •Patients do not come to the clinic until they develop severe symptoms and complications •Hospital administration has set special cardiac clinic day
Would you consider HF a key health condition of concern for the clinic and/or community?	•Limited cardiac services available and many patients in the community have not been reached	•Most patients managed in the clinic reported with heart failure at baseline
Are there any other core health issues of concern at the clinic or the community?	•Lack of family support worsened by stigma and poverty •Wide catchment area means long distances to the hospital	•Chronic conditions such as hypertension, diabetes, sickle cell disease (weekly), HIV (daily clinic), kidney disease are common cause or comorbidities of heart disease
Are there any challenges or opportunities associated with clinic or community care?	•Cardiac clinic operates autonomously with little or no intrusion from the hospital top management •Concern on continuity of the program once adapted •Clinic needs weigh scale & blood pressure machine •Essential medicines & laboratory reagent stockouts due to procurement gap •Patients cannot manage financial burden that comes with heart disease	•Staff desperately need in-service training in the current medical practice for heart health •Program could address failure to attract and retain clinical specialists affects full-time patient care delivery •Supply of essential drugs and the availability of basic diagnostic equipment for cardiovascular diseases is a challenge •No established drug distribution community model •Clinicians travel to lower-level units to provide support by reviewing patients

Given the contextual findings from Cycle 2, an additional validation cycle (Cycle 3) was added to re-confirm both the project priorities and the added components to the study approach. Both stakeholder groups approved of the study modifications and identified a local team member to lead the facilitation of the next study phase. The stakeholders also reviewed the I-RREACH tool interview guide (Supplementary file 5) and demographic survey to adapt these instruments to the local context. In the final engagement session (Cycle 4), stakeholders were provided a project summary describing the co-developed study objectives and methods for data collection. In parallel, the UHN team member (SW) conducted 3 training sessions with the local clinic leads (JK, JA), translator (FO) and UHI team (MN, CNM) to review and finalize the interview guide.

### Phase 2: Engagement and needs assessment

#### Participant characteristics

A total of 60 participants were involved in this phase across both sites: 30 patients, 20 clinicians, 10 VHTs. Using the data collected from the co-developed demographic survey, patient characteristics are shown below in Table 2. Between the two sites, patients had a relatively large age range from 18 to 84 years old. Patients in Gulu were younger than in Lira (mean age 32 v 53 years, respectively). The most common languages spoken were Acholi (Gulu *n* = 11) and Lango (Lira *n* = 10). However, in Lira, more patients spoke a combination of both English and their local language (*n* = 5). Across both sites, patients reported a variance in their marital status, but a relatively lower level of education and a higher level of unemployment.

**Table 2:** Patient characteristics

**Characteristic**		**Gulu**	**Lira**
Sample Size		15	15
Age (years)	Mean (SD)	32 (18.6)	53 (12.7)
	Range	18- 72	21- 84
Sex	Male	7	2
	Female	8	13
Ethnicity	Black African	10	-
	Acholi	3	1
	Luo	2	1
	Lango	-	11
	Kumam	-	1
	Itesot	-	1
Language	Luo	3	-
	Acholi	11	-
	Lango	-	10
	English & Langi	1	-
	English & Acholi	-	1
	English & Kumam	-	1
	English & Lango	-	1
	English & Luo	-	1
	Lango & Itesot	-	1
Marital status	Single	7	1
	Married	6	8
	Separated	2	1
	Widowed	-	5
# Of people living in home	Mean (SD)	8.7 (4.4)	6.4 (2.3)
	Range	20-Apr	10-Feb
Level of education	No Study	-	3
	Primary	9	8
	Highschool	5	3
	University	1	1
Level of reading and writing	Illiterate	4	6
	Some reading & writing	9	6
	Fully literate	1	3
Monthly household income (Ugandan Shilling)	Mean (SD)	45,000 (75,119)	74,867 (154,308)
	Range	0 – 300,000	0- 573,000
Employment status	Unemployed	12	9
	Self-employed	3	3
	Retired	-	3
Health issue of priority	Heart disease	9	6
	Rhematic heart disease	3	-
	Heart failure	2	4
	Hypertension	1	5
Self-reported health status	Poor	4	4
	Fair	3	9
	Good	8	2
	Excellent	-	-
Level of physical activity	Poor	2	6
	Fair	2	7
	Good	10	2
	Excellent	-	-

#### Survey findings

A total of 26 variables were collected through the patient demographic survey. Across both sites, patients travelled significant distances to access care, but patients in Gulu on average traveled double the distance of Lira patients (18.2 km v. 9.8 km, respectively) (Table 3). The main source of transportation was using motorbikes (Gulu *n* = 11(73%); Lira *n* = 12 (80%)). Caregiver support varied among both populations, but with a relatively younger patient demographic in the Gulu community, parents were the most common caregiver (*n* = 5). Among both patient groups, weight scales and blood pressure (BP) cuffs were rare outside the clinic, as only one patient had a BP cuff at home.

**Table 3:** Patient demographic survey–access to care resources

**Characteristic**		**Gulu**	**Lira**
Sample Size		15	15
Distance travelled to access care (km)	Mean (SD)	18.2 (15.2)	9.8 (11.1)
	Range	Jan-60	0.5—43
Type of transportation to access care	Motorbike (%)	11 (73)	12 (80)
	Motorbike/car (%)	1 (7)	-
	Taxi/Boda Boda (%)	2 (13	1 (7)
	Motorbike/bicycle (%)	-	1 (7)
	Bicycle (%)	1(7)	1 (7)
Access to caregiver support	Spouse (%)	3 (20)	3 (20)
	Child (%)	2 (13)	4 (27)
	Parent (%)	5 (33)	1 (7)
	Other (Self (%)	2 (13)	-
	Other (Brother) (%)	3 (20)	-
	Other (Grandchild) (%)	-	2 (13)
	Multiple (%)	-	5 (33)
Access to weight scale outside clinic	Yes (%)	-	-
	No (%)	15 (100)	15 (100)
Access to blood pressure cuff outside clinic	Yes (%)	1 (7)	-
	No (%)	14 (93(	15 (100)

Regarding technology usage, all patients had access to a mobile phone at home, with these largely being non-smartphones (Gulu *n* = 13; Lira n = 14) (Table 4). A variance in the reliability of cellular coverage was reported, but MTN was found as the main cellular provider. Each population had access to different community resources, but radio talk shows were identified as the main source for health information (Table 4). In Lira, with an older patient demographic, patients were found to be more engaged in cultural activities compared to the patients in Gulu.

**Table 4:** Patient Demographic survey – technology usage & community resources

**Characteristic**		**Gulu Mean (%)**	**Lira Mean (%)**
Sample Size		15	15
Access to mobile phone	Yes (at home)	15 (100)	14 (93)
	Yes (borrow)	-	1 (7)
	No	-	-
Type of mobile phone	Smartphone	1 (7)	1 (7)
	Non-smartphone	13 (87)	14 (93)
	Both	1 (7)	-
Reliability of cellular coverage	Poor	5 (33)	2 (13)
	Fair	2 (13)	5 (33)
	Good	7 (47)	7 (47)
	Excellent	2 (13)	1 (7)
Main cellular provider	MTN	10 67)	13 (87)
	Airtel/MTN	4 (27)	1 (7)
	All	1 (7)	-
	Unsure	-	1 (7)
Community resources for health information or care support	Radio talk show	7 (47)	7 (47)
	Village program	4 (27)	2 (13)
	Community Elder	1 (7)	-
	Radio & Village program	1 (7)	-
	Radio & Newspaper	1 (7)	-
	Radio & VHT	-	2 (13)
	Radio & Church	-	1 (7)
	Community gatherings	-	2 (13)
	LCI Chairperson	-	1 (7)
	None	1 (7)	-
Participation in cultural or community activities	Clearing water source	2 (13)	2 (13)
	Traditional marriage ceremonies	1 (7)	-
	Prayer group	1 (7)	-
	Cultural meeting & traditional marriage	-	2 (13)
	Cultural meeting	-	1 (7)
	Yes (not specific)		
	None	-	2 (13)
		-	2 (13)
		12 (80)	5 (33)

#### Interview findings

Five dominant themes were identified: (i) Disconnected journey of heart health management; (ii) Overburdened clinics lead to fragmented approaches to care; (iii) Strength of community support is built on trust and mutual values; (iv) Collaborative efforts can empower patient self-care; and (v) Bridging community and clinic through digital health.

- (i) Disconnected journey of heart health management

Within the remote communities, heart health was often not prioritized, as the community viewed individuals diagnosed with heart conditions as helpless. Due to this stigma, patients would only seek care support once their symptoms became intolerable, leaving the process of diagnosis to be very abrupt. In many cases, clinicians indicated that patients would come to clinic when they were already in HF, as they did not want to accept the reality of their health status.

“Most of these people are heartbroken because they find heart as the medium for life and if it has any problem, they feel helpless and they need close attention, as they tend to isolate themselves… Most people lose hope on the person affected by a heart problem, and they also abandon that person” (Gulu VHT)

“I started finding out that I am suffering from the rheumatic heart disease in the month of May. I tried to move a short distance, like from home going to a trading centre, but on my way back, I felt tired, and my hands were like all swollen, hot, and white in colour.” (Gulu Patient)

Once a diagnosis was obtained, patients were found to rarely come back for clinic follow-ups, as there was no incentive in managing their condition further. In many cases, the lack of care continuity within these populations was attributed due to the poor patient-clinician relationship. Even with clinicians providing a diverse model of care, including weight management, nutrition, and counseling, some patients were fearful in sharing the details of their symptoms because of the unfriendly response they may receive.

“The model of care really involves more medicines, counseling, giving them nutrition education, and encouraging them to do exercises and rest to when they have some issue” (Lira Clinician)

“Most patients are so fearful to express their feelings” (Gulu VHT)

“They believe medical workers have bad attitudes, they are abusive, they are not welcoming” (Lira Clinician)

Given that many patients were from low-income households, several financial constraints limited their ability to self-care. Specifically, all patients found that the cost of medication was unaffordable. Some patients indicated that they often skipped taking their medication, and would instead rest when they experienced any symptoms, due to cost-related factors. Recognizing that patients often purchased medications at the clinic pharmacies, the cost of travel also served as a major barrier to HF management. Patients expressed that they not only had to coordinate the mode of travel (e. g., bus, motorbike, walk), but they also had to prepare for the physical toll it may have on their health. With these conditions, patients felt that the benefits of clinical care were not enough to combat the physical and financial cost of managing their condition.

“Yes, I have problems- first of all the cost of the medication is high and at times I don’t have money for the medicine. And when there is no money to buy medicine, you are forced to skip taking the medicine… I normally struggle but sometimes I don’t have much capability [financial] to meet the demand for my medication. Sometimes when I am experiencing pain in the chest, I stop working and do nothing and wait for it to subside” (Gulu Patient)

“There is always the problem of money. Sometimes if I report to Mulago [National Referral Hospital], especially last time when the doctor referred me to Mulago, there was no transport. I had to struggle and spend a night at the bus park because I had no relative there, so there was problem of transport because of lack of money” (Gulu Patient)

To reduce the physical challenges associated with a patient’s condition, clinicians encouraged caregivers to help with any labour-intensive tasks in the home setting. However, with many caregivers working different jobs to cover the cost of medication, they were limited in their ability to play this supportive role. It is important to recognize that among female patients, tasks associated with domestic work were regularly expected regardless of the severity of their condition, creating a significant challenge when managing their symptoms.

“No one helps me because even my husband says I must struggle to get some money for my medication… Because I have lots of domestic work. If you are in your home and married, you have to work hard and there is no one to help you. You fetch water, wash clothes, all this makes you very tired” (Lira Patient)

“The saddest thing is when you are alone, you feel very sad. If I had someone to at least help me, it would at least be easier. I also get very tired” (Gulu Patient)

- (ii) Overburdened clinics lead to fragmented approaches to care

Within both clinical settings, clinicians expressed that the lack of bed space, equipment, and clinical expertise available, limited the quality of their care delivery. Most patients travelled far distances to be seen in the clinic, but with the significant patient volume and the inconsistent number of clinicians available, patients were left waiting in long lines for limited clinical investigation. In Lira, compared to the OPD clinic, nurses in the RHD clinic were handling more than double the caseload with half the staff, as they received referrals from both the emergency department and the OPD.

“The problem of our space [RHD clinic], we don’t have consultants coming around and even other medical officers don’t come around to the clinic. Even other interns tried but e they could not tolerate the squeezed space, so it leaves the work to all of us” (Lira Clinician)

“The clinic is overwhelmed with the numbers of patients and they [patients] also spend a lot of time waiting in the line… They [patients] come from long distances, as it is also not easy to get the expertise” (Gulu Clinician)

Despite the limited staff available in the clinic, clinicians indicated that they still failed to collaborate with VHTs. Within the community, VHTs were already providing patient counselling and facilitating patient referrals, yet they were underutilized in the clinic due to their limited training. Clinicians instead had a strong desire for an increased collaboration with the cardiologists at UHI, but the reality was that the clinic only connected with UHI for specialized consultations.

“We collaborate with nursing assistance, clinical charge physician but not the VHT and traditional healer” (Gulu Clinician)

“We were trained how to help our people. For instance, the ones suffering from various sicknesses like malaria, we give them drugs; the ones with heart problems, and those living with HIV/AIDS, we counsel in the community. Whatever we can’t handle, we refer to the hospital” (Gulu VHT)

To improve the delivery of care, participants reported there was a need for basic equipment in the clinic and advanced training for the clinicians and VHTs. One VHT suggested that with an increase in nursing support, a nurse could work alongside the VHTs in the community to provide local follow-up care. The use of collaborative linkages between heart institutes or regional health centers was also recommended, as this would help facilitate more informed clinical discussions between UHI and the Regional Referral hospitals.

“They should increase the number of nurses so that they could move with the VHTs to check on the patient” (Lira VHT)

“There should be a heart institute in the region, if we could have proper linkages direct with the patient in the village” (Gulu Clinician)

- (iii) Strength of community support built on trust and mutual values

With access to care posing a significant challenge for the remote communities, patients found sources of community support to be more reliable and efficient than clinical care. In some cases, patients indicated there was a level of distrust with non-local researchers or clinicians, as care support would only be offered for specific time periods. Once a project or collaboration expired, patients would be left with a sense of false hope and discouragement. Conversely, in the community, patients would be provided with a continuous level of support from family, neighbours, and the Church to help ease the process of care management. Patients even mentioned that they found comfort in hearing stories from members of their community on how other individuals managed their heart health within their current lifestyle.

“They take good care of me; the church members always pray for me, and they call me for services. My neighbours also try their best so that I don’t stay home alone. They say if I am bored, I will be stressed ” (Lira Patient)

“In the community you may find people who also had family members who also suffered from the same [condition], so they counsel you and give you advise to follow your prescription” (Gulu Patient)

Given the preference for in-community support, VHTs played a pivotal role in supporting patients, as they were often the initial touchpoint for care management. Despite their limited training, VHTs had a more open and trusting relationship with patients, allowing them to speak freely regarding their health and wellness. Within this relationship, patients also valued the VHTs strength and determination to support their local community, as they would volunteer their time to follow-up with patients and facilitate clinic referrals without compensation.

“VHTs are free with their patients, they interact freely with them. For instance, with a person with a heart disease to know what is going on in their lives. If we are rude to them, they will not open up to share their ideas” (Gulu VHT)

“So, when they got to know that I am sick, they [VHTs] are the ones who got me and took me to the hospital. Of course, in any hospitals you have to line up for registration, they helped me, they got me in line and gave me a number” (Gulu Patient)

Recognizing the complexity of HF management and the difficulties associated with providing culturally informed care, the VHTs experienced a number of challenges when facilitating their role in the community. Specifically, with the lack training and knowledge on HF management, many VHTs were limited in their ability to provide care support. Logistical challenges related to travelling far distances also posed a significant obstacle, as VHTs were working in the community without basic equipment, such as boots, gloves and phones.

“We have limited knowledge of the sickness, the community may ask you what should be done but you have no idea on what should be done. We refer them early enough to hospital, but we should be given phones for reliable communication and protective gears like gumboots, tags for identification… If possible, you should take your time and teach us how to manage our patient on how to come to the hospital early” (Gulu VHT)

“Another challenge is the distance we have to travel on foot to visit patients. We have to leave something [finances] to support the welfare of our families, yet we volunteer our time to help the patient… You have to pay for them [clinical care] but for us VHTs, we do it for free” (Gulu VHT)

While the importance of supporting the VHT role was evident, the community had mixed views on the use of traditional healing. Some patients turned to healers as their first source of care guidance before attending clinic, whereas others would seek herbal remedies because they could not afford the cost of transport or medication. Given the clinicians’ view that traditional healers would frequently misguide patients, there was a need for patient education to integrate both clinical and herbal approaches for heart health management.

“Sometimes they [patients] would want to go to traditional healers first before they come to hospital, as they hold them [healers] in high regard and usually they don’t want you to challenge them” (Lira Clinician)

“They [patients] are always misled by traditional herbalists and some by religion, but they usually [go to healer because they face] finance and transport issues among many others” (Gulu Clinician)

- (iv) Collaborative efforts can empower patient self-care

To empower patient self-care within the challenges of clinic and community support, there was a need for improved collaboration amongst the patient’s circle of care. This often included clinicians, VHTs, healers, community leaders, and/or caregivers. Many VHTs found there was a significant communication gap between clinicians and VHTs, as the VHTs felt they were always chasing the nurses to provide updates. Other issues regarding communication revolved around the variance in medication cost, where certain clinics were less expensive for the same drugs, but with a lack of collaboration with local pharmacies, clinicians were limited in the guidance on cost they could provide patients.

“There is also a communication gap between the clinicians and VHTs. For instance, I may have to attend to a patient personally if I have to advise the patient to go to the health centre” (Gulu VHT)

“I struggle to buy my medication, it is very expensive…Unless in [local private hospital] perhaps, when I go there, the same tablets are not as expensive as in the clinic” (Gulu Patient)

Within the community, many patients shared that they wanted to improve their health status to be an example for their family, but they did not have the knowledge on now to manage their condition within the constraints of their contextual environment. To help support collaborative clinical practices and empower patient self-care, providing patient education was highlighted as key. Clinicians recognized the significance of health education and their role in providing supportive guidance to better manage their condition at home.

“For me, I want to get improvement because, if I don’t get improvement then life becomes very hard with children. Because there is advice that I also give them, sometimes I’m also sad when my feet are swollen” (Lira Patient)

“They are able to manage it if we empower them especially through health education, support and they can manage themselves little” (Gulu Clinician)

In terms of local sources of engagement, radio shows were identified as a supportive avenue to provide health education and community sensitization. Given the common use of radio across the remote communities, clinicians suggested that the VHT role could be leveraged to further provide clinically informed health education within the local setting.

“They access it [heath education] through like a community dialogue organized by heath care providers, radio talk shows and maybe others from the VHTs that are trained about the problem [health condition]. They [VHTs] discuss it in the community and make community awareness and sensitization” (Lira Clinician)

“Sometimes I hear from ABS radios station. There is someone from Gulu University who sometimes talk about other herbs that can cure heart disease or heart problem” (Gulu Patient)

- (v) Bridging community and clinic through digital health

With the complexities associated with the clinic and community environment, the use of mobile phone-based solutions was viewed as a valuable opportunity to support HF care. Specifically, by expanding the use of mobile phones for clinical care, patients are able to receive care support without the burden of travel. With many clinic processes able to be completed virtually, clinicians can modify a patient’s clinical regimen before their condition significantly worsens. However, to maintain the integrity of the patient-clinician relationship, clinical examinations would need to remain in-person.

“It [digital intervention] should help me to follow up with my patient, address their medical needs at a convenient time, and help to provide a few recommendations” (Gulu Clinician)

“Yes, like management of pain, counseling can be done, one can be instructed over the phone, but examination should remain face to face and history taking” (Gulu Clinician)

Recognizing the unique barriers faced by each community, patients highlighted that the reliability of a mobile phone-based tool would depend upon mobile phone network coverage. Given that some patients shared a mobile phone with their spouse or parent, a structured regimen for when the phone was to be used was needed. Clinicians also expressed that with patients often working multiple jobs to provide an income for their family, reducing the costs associated with using the digital tool would be integral. This included minimizing the amount of airtime needed for the mobile phone-based tool, as well as creating avenues for patients to obtain care support at lower health centers.

“It’s about the place I’m staying in there is no electricity where we stay, so the phone sometimes shuts down” (Gulu Patient)

“It may be very necessary to have the system expanded in the regional referral hospital so that the patient starts accessing it at the reduced cost. It is also necessary to train the health care worker” (Gulu Clinician)

In reflection of these challenges, to help support the adoption and sustained use of a mobile phone-based program, both the features and delivery of the program would need to be customized for the local context. Specifically, features related to language and visual aids were recognised as core components to improve the usability of the tool, especially amongst patients of low health literacy backgrounds. One clinician also felt that by including a patient helpline, this would provide patients with a supportive backbone to help them throughout their care journey.

“I think all the languages if they can. Because I am not the only person with the heart disease and an Acholi of course, I am educated but there are some who are not educated and suffering from the same disease, and they have to know about that. So, it should be printed in Acholi, English and other languages. For all of us to understand” (Gulu Patient)

“Customer care help line should be there, local arrangement between patient and doctor besides English and other languages should be installed” (Gulu Clinician)

#### Interview evaluations

Using the I-RREACH tool’s participant feedback evaluation, participants at both sites reported positive responses regarding the interview session (Table 5). Some key highlights include patients valuing that the interviews were facilitated in their preferred language and that they were given the opportunity to exchange ideas about how different aspects of their life, from their comorbidities to their cultural traditions, impact their overall health and well-being.

**Table 5:** Combined participant feedback evaluation

Feedback Questions	Strongly Disagree	Disagree	Neutral	Agree	Strongly Agree
1. The questions asked were clear and made sense to me	0 (0%)	0 (0%)	3 (10%)	18 (60%)	9 (30%)
2. I think the researcher understood my perspective (two-way exchange of information)	0 (0%)	0 (0%)	1 (3.3%)	20 (66.7%)	9 (30%)
3. After attending this interview, I have a better understanding of the project goals and how the system can be used to support my care needs	0 (0%)	0 (0%)	2 (6.7%)	21 (70%)	7 (23.3%)
4. I felt comfortable with what we discussed during the interview	0 (0%)	0 (0%)	1 (3.3%)	23 (76.7%)	6 (20%)
5. The interview was a good way to exchange information and ideas related to the project	0 (0%)	0 (0%)	1 (3.3%)	23 (76.7%)	7 (23.3%)
6. What did you like best about the session?	• “Questions explained in language I understand” • “Happy about confidentiality and liked reminder of patient care • “Very comfortable and felt questions were really thinking about how to help patient” • “Liked the explanation given before the interview” • “Engages about information about community, culture and beliefs (all aspects of life)” • “Liked that system will help us talk to doctors even when in long distance” • “Gives me confidence, I like the idea and technology which is coming up to help patients like me” • “It will help me in looking after my health. It was a friendly interview. We exchanged ideas” • “Happy that we shall start getting help on the phone”				
7. Is there anything you think we should change?	•Interactive but a bit lengthy” • “Project should be expanded to other health problems”				

#### Phase 3: Design and system adaptations

To identify the design requirements for the community-based adaptation of the Medly Uganda program, five iterative co-design meetings were conducted with the stakeholders from each community (total *n* = 10). Using the findings from Phase 2 and the stakeholder co-design meetings, six design requirements were identified in relation to the study findings (Table 6).

**Table 6:** Medly uganda remote clinic design requirements

Research Finding	Design Requirement
**1. Priority of heart health minimized:** Lack of sensitization on heart health and disease management due to focus on HIV and family income generation. Community often views diagnosis of heart condition as helpless	The program will include source of education within community regarding: 1. heart health and wellness 2. common heart HF symptoms and 3. pathways to obtain care or investigation for HF symptoms both at lower health centres and specialized cardiac clinics Avenues for education include: Radio talk shows, community support groups, knowledge keepers, church events, clinic visits
**2. Clinic workflow overwhelmed due to limited staff and resources:** Clinicians and VHTs report that limited staff, infrastructure and equipment cause disruption in workflow and patient care experience. High frequency of patients often leads to long wait times and shorter clinician-patient interactions	The program will play a role in reducing the clinic workflow burden by empowering patient self-care in the remote home setting, and subsequently reducing the number of unnecessary and/or emergent patient visits to the clinic
**3. Lack of care continuity and relationship building:** Patients initial clinic visit often occurs when hospitalized or patient is in HF. Frequency of care touchpoints vary from monthly to every 6-months. However, large disconnect between visits reported, as patients have either given up on improving their condition or are fearful to express state of well-being	The program will provide continuous feedback to ensure level of care continuity is maintained throughout patient’s care journey. Feedback should reflect patient’s individual condition in relation to their reported symptoms
**4. Lack of specialist expertise available:** Nurse-led clinic, cardiologist rarely available and connection to Uganda Heart Institute (UHI) mainly occurs for patient referrals or transfers. From patient’s perspective, burden of travel to UHI often does not outweigh benefits. From clinician perspective, Medly Uganda system currently would only benefit patients able to travel to UHI to obtain HF diagnosis	The program will facilitate collaborative connection between cardiac clinic and UHI to provide more streamlined care support via: 1. UHI-led cardiac training will be provided to local clinic personnel to allow for growth and capacity development 2. UHI will provide in-person cardiologist or cardiac fellow visit every 3–5 months for mass screening events 3. Medly Uganda dashboard will be adapted to include multiple clinician and admin roles (i.e., Gulu/Lira nurse, UHI cardiologist) across different sites, as source of communication and patient review
**5.** **VHTs undervalued & underutilized:** VHTs often serve as the initial touchpoint in patients’ care journey. VHTs also provide frequent care follow-ups within the community, but they are not viewed as part of care team due to their limited knowledge on cardiac care and HF symptoms	The program will facilitate a collaborative workflow amongst clinicians and VHTs to improve the patient’s care experience via: 1. Recognized position within program 2. Direct communication channel with clinic nurse 3. Clinic-led training on heart health, HF symptoms and self-care practices, to allow for more informed patient visits 4. Structured patient follow-up guide created in collaboration with VHTs to guide VHT- patient interactions within the community
**6.** **Financial constraints limit ability to seek care:** Patients not motivated to self-care due to financial difficulties, as cost of medication and transport reported as largest barriers to disease management	The program will be free for patients to use. Active self-care promoted by the system will also reduce the financial burden of travelling long distances to obtain clinical support

First, with the need for community heart health education, a program orientation guide and patient education manual have been developed to allow patients to better understand the purpose of the program and how HF should be managed within the context of their local community (Table 6). Second, given the challenge of limited staffing, the program will focus on empowering home-based self-care to reduce the number of in-person clinic visits needed. Third, to create a level of care continuity and motivate patients in maintaining in their care routine, they will be provided personalized self-care advice in relation to their reported symptoms directly on their mobile phone. Fourth, with the lack of specialist support in the remote communities, the Medly Uganda program will support a collaborative linkage between UHI and each local clinic. Specifically, UHI will provide cardiac training to the local staff and will provide an in-person cardiologist visit every 3–5 months to support a mass screening event. Next, to integrate VHTs as direct members of the care team, they will be provided with 1) a recognized position within the Medly Uganda program, 2) direct communication line with the clinic nurse, 3) clinic-led training on HF care and self-care practices, and 4) a structured patient follow-up guide. Finally, with financial burden of HF management, the last design requirement will focus on reducing the costs of patient care, by allowing the program to remain free or at minimal cost for patient usage.

With the envisioned role of VHTs within the Medly Uganda program, an additional three co-design meetings with the VHTs were held at each site (total *n* = 6). The two-eyed seeing method was leveraged throughout each meeting to ensure the VHTs operational responsibilities were customized reflection of the clinical needs of the program and the local dynamics of each community. Following the completion of the co-design discussions, the VHTs agreed on facilitating two primary roles within the Medly Uganda program: 1) in-community follow-ups and 2) medication delivery (Supplementary file 4: Appendix D). Given the burden of travel and the need for care continuity, VHTs felt they would be able to provide in-community follow-ups on an as-needed basis and would set weekly to biweekly visits for patients who required further support. However, recognizing the differences between the Gulu and Lira community dynamics, the VHTs in Gulu indicated that they found travelling to clinic weekly burdensome, and instead opted to elect 1–2 Lead VHTs to conduct the weekly clinic visits and medication pick-ups. Through this role, each VHT would still conduct their in-community follow-up visits, but they would deliver their patient notes each week to designated Lead VHT for clinic drop off. Conversely, in Lira, VHTs preferred to have each individual VHT conduct the weekly clinic visits and medication drop offs, as each parish had their own dedicated responsibilities. To help facilitate the VHT role and support a more systematic process for the in-community visits, a VHT booklet was co-developed with each community. This booklet includes two primary components 1) patient interaction guide with questions related to the HF symptoms, medication adherence, local diet, follow-up care and housing, and 2) contact information for Lead VHT, VHTs in district, patients, and clinic.

## Discussion

### Principal findings

In Uganda, the inequitable distribution of health resources has led rural communities to experience a significantly higher level of illness compared to urban communities [15]. With the burden of HF continuing to grow and conditions of rural poverty worsening, there is a need for context-specific care solutions to be introduced. Recognizing the increasing use of mobile phones across Uganda, digital health programs, such as Medly Uganda, can be utilized to help improve access to HF care [18, 19]. However, with rural communities facing significantly different contextual challenges than urban settings, various cultural, social, and structural factors would need to be integrated within the design of a digital therapeutic program. With this, to better understand the unique barriers and local facilitators influencing HF outcomes, this study worked in partnership with the remote communities in Gulu and Lira, to adapt the Medly Uganda program to their local needs and capabilities.

Across both rural populations, clinic and community stakeholders signified that a mobile phone-based HF program could help support improved access to care for their patients. Importantly, to prevent the cycle of failed pilot projects from re-occurring, the success of the Medly Uganda program would be dependent on its ability to provide cardiac care support from both UHI and local community resources [5]. Recognizing the lack of specialist expertise in the Northern clinics, this study has facilitated a series of commitments from UHI to provide sources of education and training. To better empower local community resources, VHTs will also be provided a dedicated position within the Medly Uganda program to support in-community follow-ups and medication delivery. By integrating the VHT role within both the clinical and community components of the Medly Uganda program, this model of care directly aligns with the MOH’s vision for VHTs to bridge the gap between communities and health facilities [33]. Given the potential benefits of this care model, the findings from this study can be used to help contextualize the VHT role to support other chronic conditions. For example, in one study the use of multi-month dispensing (MMS), where a patient receives > 3 months of medication refills, was used to reduce the burden of travel for patients with hypertension [37]. Despite patients receiving MMS displaying improvements in hypertension control, the use of MMS was limited to patients who were stable [37]. With the integration of VHT-led medication delivery and in-community follow-ups, patients of varying levels of health status would be able to benefit from this model of care, as clinicians could modify their medication without the need for a clinic visit.

Beyond the need for increased clinical capacity, factors related to poverty and the disparities in a population’s SDH, led income generation and housing to be of higher importance than heart health management. The interplay between poverty and health care access has also made patients less likely to attend follow-up visits, as they were travelling up to 19 km by motorbike for clinic visits (Table 3). In many cases, patients were often referred to UHI to receive specialist care, but the cost of travel outweighed the uncertainty of improved health outcomes. Given the financial and physical burden of HF management, one benefit to the Medly Uganda program is the provision of personalized HF care directly to patients at a lower cost. We recognize that the use of the Medly Uganda program may lead to accumulated costs around electricity and Airtime consumption, thus we will seek to understand the specifics of cost associated with this program in an upcoming study. By leveraging the VHT role coupled with the use of patient education manuals and community sensitization initiatives, this study envisions that the Medly Uganda program will be able to empower HF self-care and community wellness. However, it is important to recognize that despite the program’s ability to bring care services closer to home, many essential medications often fluctuate in both their availability and cost, leaving medication access to remain a significant challenge [38] With this barrier, future research should work to characterize the cost and demand for specific medications to better support patient care management and the financial sustainability of the program.

While recognizing that each rural population faces unique barriers that influence their health and well-being, this study integrated a series of reflexive methodologies based on Western and Indigenous research paradigms to better understand the significance of each local environment. The lived experiences and unique perspectives of each clinical and community stakeholder were used to guide all program adaptations, whereby additional participatory consensus cycles and co-design workshops were added to further contextualize the design requirements. Specifically, within the development of the VHT role, the Gulu and Lira sites present different structures for the service of medication delivery and in-community follow-ups to better suit the preferences of the local community. Community views on traditional medicine were also explored, whereby patients were found to often turn to herbal remedies when conventional medicines became unaffordable [39]. Ultimately, by working in partnership with stakeholders beyond the clinical team, this study has worked to help bridge both Western and Ugandan worldviews regarding the management of heart health and wellness.

### Limitations

There are a number of limitations to this study. First, participants recruited in this study all had a direct linkage with either the Gulu Regional Referral Hospital or Lira Regional Referral Hospital. Outreach to the surrounding lower regional health centers and community groups were initiated, but with the restrictions associated with the COVID-19 pandemic, many individuals declined to participate, as they were not comfortable travelling to the clinic site. Second, despite the use of the two-eyed seeing approach, whereby at least one Ugandan team member would co-analyze the study data, the UHN researcher (SW) was unable to fully experience the lived insights of the local context with the absence of an in-person community visit. SW initially aimed to travel to Uganda to facilitate all project activities in-person, however, this approach was modified to accommodate for the travel restrictions and public health guidelines posed by the COVID-19 pandemic. To mitigate this challenge, weekly team meetings were held with each stakeholder team and the UHN researcher virtually attended all participant interviews through the Zoom platform. Lastly, with community members indicating mixed views on traditional medicine, this study recognizes that healers were not recruited for study participation, leaving our knowledge on traditional medicine to be limited by the feedback provided from the various participant groups. Engagement of traditional healers represents an important component of our future work.

## Conclusion

As the incidence of rural poverty continues to grow in Uganda, to facilitate meaningful change, health services need to recognize the unique circumstances, challenges, and experienced barriers that effect an individual’s well-being. A central aim of this study was to honor the traditions and strengths of the local community to better understand the realities of HF management. By integrating a community-based approach within the UCD process, six core design requirements were identified to adapt the Medly Uganda program for the Northern community context. Despite the burden of travel being recognized as the largest barrier for care, by customizing the delivery of the Medly Uganda program and empowering the use of trusted local resources, this will help enable culturally informed care closer to home.

### Supplementary Information

**Additional file 1.** I-RREACH clinic profile and community demographic survey.

**Additional file 2.** Adapted I-RREACH Feedback Survey.

**Additional file 3.** Patient Demographic Survey.

**Additional file 4.** Appendix A-D.

**Additional file 5.** Interview Guide.

## Data Availability

The data supporting the findings of this study are available from the corresponding author upon reasonable request. Program design requirements are appended as Supplementary Data.
