# Supplementary material for: Bridging community and clinic through digital health: Community-based adaptation of a mobile phone-based heart failure program for remote communities in Uganda
Source: BMC Digit Health. 2023 Jun 16;1(1):20. doi: 10.1186/s44247-023-00020-5 (PMC11116269; doi:10.1186/s44247-023-00020-5)
Supplement: Supplementary file 1 — Additional file 1. I-RREACH clinic profile and community demographic survey. [file 44247_2023_20_MOESM1_ESM.docx]

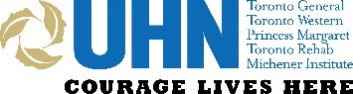

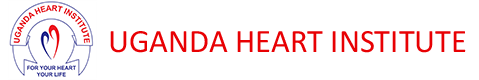

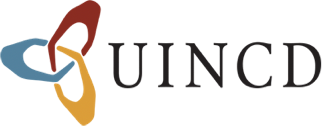


**Medly Uganda: Mobile phone-based program for remote heart failure care, the Uganda Heart Institute Remote Clinic Outreach Initiative**

**Clinic Engagement Session Guide & Checklist**

In collaboration with

*University Health Network*

Toronto. Ontario

and

*Centre for eHealth Innovation*

Toronto, Ontario

and

*Uganda Initiative for Integrated Management of Non-Communicable Diseases*

Kampala, Uganda

and

*Uganda Heart Institute*

Kampala, Uganda

**Clinic Profile**

To obtain a better understanding of the current clinic operations, please provide responses to the below questions. If the questions do not apply, simply input, “not applicable.”

1. Address and location of clinic space(s):
2. Who is the current health care lead at the clinic?
3. Who is the current physician lead at the clinic?
4. What type of patients are mostly seen in the clinic? (i.e., heart failure, rhematic disease?
5. How many patients are currently seen at clinic?
6. Do patients face any specific challenges that limit them from coming to the clinic?
7. What is the structure and leadership of the clinic nursing staff?
8. Does the clinic face any challenges with resourcing or staff availability?
9. Are there any current efforts to support cardiac health at the clinic?
10. Are there any health registries and/or databases currently part of the clinic?
11. Who is the first point of contact for help for person with cardiac care:

**Community Profile**

To obtain a better understanding of the current community context, please provide responses to the below questions. If the questions do not apply, simply input, “not applicable.”

1. Name of Community:
2. Community website (if applicable):
3. Is there a current community leader? (name and role)
4. Are there any current efforts to promote health or wellness, such as programs or community centres?
5. Are there any present health champions or decisions makers present in the community (names and role)?
6. What are the current health issues of priority in the community?
7. Who is the first point of contact to help a person with cardiac care in the community?
8. Are there any programs available to help with chronic disease management programs/assessments recently set up in community?
9. How reliable does the community current find cellular coverage?
10. What is the main cellular service provider(s)?
