# Supplementary material for: Bridging community and clinic through digital health: Community-based adaptation of a mobile phone-based heart failure program for remote communities in Uganda
Source: BMC Digit Health. 2023 Jun 16;1(1):20. doi: 10.1186/s44247-023-00020-5 (PMC11116269; doi:10.1186/s44247-023-00020-5)
Supplement: Supplementary file 2 — Additional file 2. Adapted I-RREACH Feedback Survey. [file 44247_2023_20_MOESM2_ESM.docx]

**Intervention and Research Readiness Engagement and Assessment of Community Health Care (I-RREACH) feedback form**

| **Your feedback would be appreciated regarding the interview sessions for the Medly Uganda project. We would appreciate you taking the time to complete this evaluation form and returning it at the end of the interview.** | | | | | |
| --- | --- | --- | --- | --- | --- |
| **Content evaluation (check one only)** | | | | | |
| 1. The questions asked were clear and made sense to me. | **Strongly Disagree** | **Disagree** | **Neutral** | **Agree** | **Strongly Agree** |
| 2. I think the researcher understood my perspective (two-way exchange of information) |  |  |  |  |  |
| 3. After attending this interview, I have a better understanding of the project goals and how the system can be used to support my care needs |  |  |  |  |  |
| **Cultural safety evaluation**  4. I felt comfortable with what we discussed during the interview  Please Explain: |  |  |  |  |  |
| 5. The interview was a good way to exchange information and ideas related to the project.  Please Explain: |  |  |  |  |  |
| 6. What did you like best about the session?  Please Explain: |  |  |  |  |  |
| 7. Is there anything you think we should change?  Please Explain: |  |  |  |  |  |
