# Supplementary material for: Bridging community and clinic through digital health: Community-based adaptation of a mobile phone-based heart failure program for remote communities in Uganda
Source: BMC Digit Health. 2023 Jun 16;1(1):20. doi: 10.1186/s44247-023-00020-5 (PMC11116269; doi:10.1186/s44247-023-00020-5)
Supplement: Supplementary file 3 — Additional file 3. Patient Demographic Survey. [file 44247_2023_20_MOESM3_ESM.docx]

**PATIENT DEMOGRAPHIC SURVEY**

| Patient ID |  |
| --- | --- |
| Patient Initials |  |

**Patient Information (to be completed by PATIENT)**

| Age [*years*] |  |
| --- | --- |
| Sex [*male or female*] |  |
| Ethnicity |  |
| Language |  |
| Marital status [*single, married, divorced, widowed, or separated*] |  |
| Number of people living in home |  |
| Level of education [*no study, primary, high school, or university*] |  |
| Level of reading and writing [*illiterate, some reading and writing, or fully literate]* |  |
| Monthly household income [*USD*] [*Shillings]* |  |
| Employment [*unemployed, employed, self-employed, or retired*] |  |
| Self-reported health status [*poor, fair, good, or excellent*] |  |
| Level of physical activity [*poor, fair, good, or excellent*] |  |
| Access to caregiver support [*spouse (wife/husband), child (son/daughter), friend, other, or none*] |  |
| Distance travelled to access care [*km*] and  care centre commonly accessed [*Clinic, nursing station, health centre, or hospital*] |  |
| Type of transportation to access care centre [*car, public transportation (bus, taxi), motorbike*] |  |
| First contact person to provide care [*lower health facility staff, village health team (VHT), nurse, or clinician*] |  |
| Health issue(s) of priority [*heart failure, heart disease, diabetes, hypertension, other*] |  |
| Access to weight scale outside of clinic [*yes (at home), yes (from a friend), yes (other), or no*] |  |
| Access to blood pressure cuff outside of clinic [yes *(at home), yes (from a friend), yes (other), or no*] |  |
| Access to a mobile phone [yes *(at home), yes (borrow from a friend), yes (other), or no*] |  |
| Type of mobile phone (if applicable) [*smartphone or non- smartphone*] |  |
| Main cellular service provider used (if applicable) [*MTN, Airtel, Africell*] |  |
| Reliability of cellular coverage [*poor, fair, good, or excellent*] |  |
| Community resources for health information or care support [*radio talk show, village programs, community Elder]* |  |
| Participation in cultural or community activities (if applicable) |  |

**If there are any other comments or information you would like to add please fill in the section below:**
