# Supplementary material for: Bridging community and clinic through digital health: Community-based adaptation of a mobile phone-based heart failure program for remote communities in Uganda
Source: BMC Digit Health. 2023 Jun 16;1(1):20. doi: 10.1186/s44247-023-00020-5 (PMC11116269; doi:10.1186/s44247-023-00020-5)
Supplement: Supplementary file 4 — Additional file 4. Appendix A-D. [file 44247_2023_20_MOESM4_ESM.docx]

**Appendix A. I-RREACH participatory consensus cycle activities and output**

|  | Engagement Session Activity | Engagement Session Output |
| --- | --- | --- |
| Cycle 1: Initiation | - Clinic & community stakeholders were invited for a virtual engagement session to introduce the purpose for the collaboration - Findings from previous scoping reviews used to guide discussion on current care challenges present in community | - Focus on heart failure and rhematic heart disease - Emphasis on Medly Uganda program supporting improved collaboration with UHI cardiac specialists - Both sites completed adapted I-RREACH clinic profile and community demographic survey - Integration of Village Health Teams to expand community outreach |
| Cycle 2: Validation | - In-person site visit conducted to obtain a deeper understanding regarding the local environment - Summary of program objectives given to all stakeholders using virtual member checking | - Both sites completed community profile tool - Co-developed patient journey map showcasing current clinic processes, care challenges and Medly Uganda program opportunities |
| Cycle 3: Validation | - Additional cycle using virtual engagement session to explore added components to study approach | - Identified local team member at each clinic to conduct interviews in local language and allow UHN researcher to connect virtually - Co-developed I-RREACH interview guide and patient demographic survey |
| Cycle 4: Finalization | - Final member checking with decided study objectives through a written feedback invitation - Stakeholders completed questionnaire for input on logistical aspects of study | - Conducted 3 training sessions with nurses and translators to review/ revise interview guide - Clinic and community recruitment strategy incorporated |

**Appendix B. Adapted I-RREACH Clinic Profile Survey**

| Clinic Profile Question | Gulu Clinic | Lira Clinic |
| --- | --- | --- |
| Location of clinic space: | Gulu Regional Referral Hospital | Lira Regional Referral Hospital |
| What type of patients are mostly seen in the clinic? (i.e., heart failure, rhematic disease? | Rheumatic Heart Disease and Heart Failure | Rheumatic Heart Disease and Heart Failure |
| How many patients are currently seen at clinic? | New patients - Daily 5-10 patients and Monthly 150-180 patients | Over 200 Rheumatic heart disease; limited data to estimate patients in OPD |
| Do patients face any specific challenges that limit them from coming to the clinic? | 1. Lack of transport 2. Limited and inconsistent drug supply 3. Lack of proper family support | 1. High transport costs to reach clinic, as clinic covers > 7 surrounding districts within Northern region 2. Most patients require referral letter to obtain access to hospital and others are self-referrals 3. Long wait times - clinicians attend to both in-patients and OPD clinic 4. Inconsistent/lack of clinician/ cardiologist support 5. No cardiac medicines available at the facility |
| What is the structure and leadership of the clinic nursing staff? | Research Nurse, Lead Nurse, Injection Nurse | Lead Research Nurse coordinates site and another research nurse with whom they manage the clinic |
| Does the clinic face any challenges with resourcing or staff availability? | Yes: Research Nurse currently handles all roles with the help of the data assistant | Yes: Limited staff availability at OPD cardiac clinic. |
| Are there any current efforts to support cardiac health at the clinic? | Expect Government hospital to provide more human resource (i.e., nurses and doctors) | Hospital has tried to adjust budget to cater for some cardiac medicines needed by patients. |
| Are there any health registries and/or databases currently part of the clinic? | Yes: RHD registry and OPD register | Yes: RHD Registry |
| Who is the first point of contact for help for person with cardiac care? | Nurse | Research Nurses |

**Appendix C. Adapted I-RREACH Community Demographic Survey**

| Community Demographic Question | Gulu Clinic | Lira Clinic |
| --- | --- | --- |
| Are there any current efforts to promote health or wellness, such as programs or community centres? | Radio talk shows | Radio talk shows |
| Are there any present health champions or decisions makers present in the community (names and role)? | Community Health Workers (CHWs)/ Volunteer Health workers sensitise the community and do refer patients for care | Village Health Teams (VHTs) help with referral of community case |
| What are the current health issues of priority in the community? | 1. Lack of awareness and sensitization 2. Limited health facilities 3. Low staffing levels 4. Lack for medicines in low level health facilities | 1. Access to health care |
| Who is the first point of contact to help a person with cardiac care in the community? | Nurse and CHWs | VHTs and lower health facility staff |
| Are there any programs available to help with chronic disease management programs/assessments recently set up in community? | Not applicable | None |
| How reliable does the community current find cellular coverage? | Relatively covered with some place lacking network | Some communities have poor network, but most have cellular coverage |
| What is the main cellular service provider(s)? | MTN and Airtel | MTN, Airtel and Africell |

**Appendix D. VHT role within adapted Medly Uganda program**

| VHT Role/ Resource | Program Considerations |
| --- | --- |
| In-community follow-ups | - With each village having dedicated VHT responsible for overseeing patients, the system needs a directive process to allow patients from specific villages to be seen by VHT of same village - VHTs require training and logistical support (i.e., transport costs, rainboots, notebook) - VHTs spend a significant amount of time to trying to locate a patient for their follow-up. VHTs need a booklet or source of information with the patients’ contact info to better facilitate in community visits |
| Medication delivery | - Medication delivery will depend on willingness of patient to consent to process - Gulu VHTs indicated travelling to clinic weekly to drop off follow-up notes can be burdensome, and instead agreed on identifying 1-2 Lead VHTs to oversee weekly clinic visits   - Each VHT will conduct in-community follow-up visits but will deliver their notes for each week to designated VHT for clinic drop off   - Patient medication drop-off will follow same routine, where each VHT will also provide the prescriptions attached to their follow-up documents for the Lead VHT to conduct clinic visit - Lira VHTs would prefer to have individual VHTs pick up medication and conduct follow-up, as each parish has own dedicated responsibilities for their community |
| VHT Booklet | 1. Contact information:    - Lead VHT contact info (name, number, address)    - VHT list with associated village and contact number    - Patient list with contact numbers (*solely for Lead VHTs)    - Clinic information (address, nurse number, type of services provided) 2. Patient interaction guide with template forms - question/answer checklist with option for medication pick-up    - HF signs & symptoms    - Medication regimen – when to take drugs, where to obtain drugs    - Common side effects & Education for patients on medication purpose    - Nutrition – what foods are available, when are meals consumed,    - Follow-up visits – when can patients be seen again/where    - Program enrolment – how can others join and for how long will it be available    - Housing – where are they staying, is it safe?   * Bottom of template form will have notes section to add comments on condition/treatment concerns or need for clinician review to allow changes to clinical regimen to be made on a preventative basis |
