# Supplementary material for: Bridging community and clinic through digital health: Community-based adaptation of a mobile phone-based heart failure program for remote communities in Uganda
Source: BMC Digit Health. 2023 Jun 16;1(1):20. doi: 10.1186/s44247-023-00020-5 (PMC11116269; doi:10.1186/s44247-023-00020-5)
Supplement: Supplementary file 5 — Additional file 5. Interview Guide. [file 44247_2023_20_MOESM5_ESM.docx]

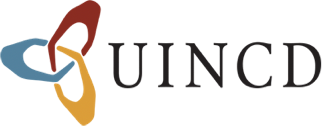

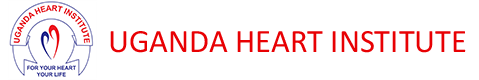

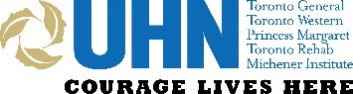


**Semi-Structured Interview Guide** -

Patient

**Study Title** Medly Uganda: Mobile phone-based program for remote heart failure care, the Uganda Heart Institute Remote Clinic Outreach Initiative

**Principal Investigator (PI):** Dr. Joseph Cafazzo, PhD, PEng

Executive Director, Centre for eHealth Innovation

Phone number: +1 416 340-4800 x4765

[Joe.Cafazzo@uhn.ca](mailto:Joe.Cafazzo@uhn.ca)

**Local PI:** Dr. Isaac Ssinabulya, MBChB, MMed

Co-Director, Uganda Initiative for the Integrated Management of Non-Communicable Diseases (UINCD)

Medical Officer Special Grade Uganda Heart Institute

Phone number: +256 782 083968

[ssinabulyaisaac@gmail.com](mailto:ssinabulyaisaac@gmail.com)

**Co-Investigators:**  Dr. Heather Ross, MD, MHSc, FRCPC

Division Head, Cardiology, University Health Network

[Heather.Ross@uhn.ca](mailto:Heather.Ross@uhn.ca)

Dr. Jeremy Schwartz, MD

Co-Director, UINCD

[jeremy.schwartz@yale.edu](mailto:jeremy.schwartz@yale.edu)

Dr. Ann Aktiteng, MBChB, MPH

UINCD Deputy Director

[akitenga@gmail.com](mailto:akitenga@gmail.com)

**Study Coordinator:**  Sahr Wali, MSc

PhD Student, University of Toronto

[Sahr.wali@mail.utoronto.ca](mailto:Sahr.wali@mail.utoronto.ca)

**Version Date:** April 6^th^, 2021

**Interview Guide: Patient**

**Introduction (~ 3 minutes)**

Hello [Name of Patient], my name is [Name of Interviewer], I would like to first thank you for agreeing to participate in today’s interview. The purpose of this study is to better understand how patients like you currently manage your heart health, in an effort to design a program to better support your care needs. I’d like to remind you that this interview will be recorded for research purposes, however, everything you say will remain confidential. At any point during the interview, you can also ask us to stop the interview or skip any questions you do not feel comfortable with. Today, we also have a researcher from Canada available to join the interview but would like to first get your approval and consent for them to remain present. Are you okay with the researcher joining via Zoom?

**Interview Topic 1 - Experiences with Clinic Processes & Heart Failure Management (~ 15 minutes)**

*General Experience with Clinic Care*

- When/how did you find out about your heart failure?
- Can you tell me about your experience here at the clinic?
  - How often do you have appointments or follow-ups at the clinic?
  - Do you find there are any challenges with attending your clinic appointments?
- How would you describe your relationship with your doctors and nurses?
  - Can you tell me about who helps you with your care at the clinic?
  - Can you describe to me what a typical appointment in the clinic looks like for you? (i.e., when you arrive and by time you leave)
- Where do you usually go when you need care for your heart failure or other conditions?
  - Are there other facilities that you go to for care such as lower regional health centers, traditional healer, Church or VHTs? If yes, could you describe how so?
    - - What is the purpose/reasoning for these interactions?
      - How frequently do you connect with these clinicians?
      - Are there different members part of your care team (i.e., nurses, physicians, community support, VHTs)?

*Heart Failure Management*

- How do you currently manage your heart failure condition? (i.e., self-monitoring of nutrition, weight, exercise, medication)?
  - Do you ever change your management in response to symptoms (e.g., shortness of breath or fluid buildup in legs)? If so, what do you do about it?
  - Do you have support from a caregiver at home to help with your heart failure?
  - Do you have any other challenges managing your conditions due to financial limitations? (i.e., medication costs)
- How confident are you in taking care of yourself with respect to your heart failure condition?
  - What aspect of self-care do you find the hardest?
  - Do you find any of the self-care tasks challenging, unnecessary or tiring?
  - What do you think would help you take better care of yourself with respect to your heart failure condition?

**Interview Topic 2: Community and Health Services Landscape (~ 10 minutes)**

- How much of a priority is heart health to your neighbours, friends, Church and to you?
- If you needed access to health information, where in the community would you usually go, if anywhere? (i.e., cardiologist, clinic nurse or other members of the community)
- Does the media or the radio get involved in educating the community?
- Do traditional healers get involved in health education?
- In the community, are there any local support groups or health programs to help patients, like you, with your care challenges or questions?
  - Do you use any traditional medicines or approaches to health and wellness for managing your heart failure or heart health?
  - Are there any other clinics or areas set up to help measure heart failure related symptoms such as weight or blood pressure?
    - Where are they located and who takes the blood pressures or weight?
- Do you find that there are any barriers that prevent you from being able to access these services? If so, what?
  - Are there certain beliefs in the community that prevent you from accessing care services (i.e., religious, cultural, social, economic)?

**Interview Topic 3 - Digital Tools, Technology & Virtualization (~ 15 minutes)**

*General Use*

- Do you generally use any type of mobile phone?
  - If yes: What do you usually use it for?
  - If no: Do you feel comfortable using one if available?
- How do you currently use your mobile phone?
  - What communication channels do you use most frequently (e.g., text messaging, calling, internet or mobile money)?
  - How comfortable do you feel in using your mobile device?

*Healthcare Use*

- Have you ever used your phone to track or manage your health?
  - If yes: What have you used it for and what was your experience with it (i.e., easy, understandable)?
    - What did you like or not like about it?
    - Do you still use it? Or if not, what made you stop using it?
  - If no: do you see this as something you would be comfortable using?
- How would you feel about using a mobile system on your phone to help you better manage your heart failure?
  - If yes: What would you want the mobile system on your phone to help you specifically with?
  - If no: Why not and what would potentially make you use one?
  - What are some benefits you would expect?
  - What are some challenges you would expect?
  - Are there any aspects of care that you believe could be completed over the mobile phone?
  - Are there any aspects of care that should remain face-to-face?
- How can we design the mobile phone program to make it more appealing to you?
  - What are some features you would find helpful?
    - Language
    - Local context
    - Overall design
- Are there any reasons that you think would lead you to stop using the mobile phone program for your heart failure management?

**Conclusion (~ 5 minutes)**

1. Is there anything else you would like to mention with respect to healthcare, heart failure management or technology that we didn’t get a chance to discuss?
2. Expression of gratitude for participation.
3. Explanation of next steps in the research project.
